# Pyrvinium Targets the Unfolded Protein Response to Hypoglycemia and Its Anti-Tumor Activity Is Enhanced by Combination Therapy

**DOI:** 10.1371/journal.pone.0003951

**Published:** 2008-12-16

**Authors:** De-Hua Yu, James Macdonald, Guohong Liu, Amy S. Lee, Mimi Ly, Timothy Davis, Ning Ke, Demin Zhou, Flossie Wong-Staal, Qi-Xiang Li

**Affiliations:** 1 iTherX Pharmaceuticals, Inc., San Diego, California, United States of America; 2 Department of Biochemistry and Molecular Biology, USC/Norris Comprehensive Cancer Center, Keck School of Medicine of the University of Southern California, Los Angeles, California, United States of America; University of Hong Kong, China

## Abstract

We identified pyrvinium pamoate, an old anthelminthic medicine, which preferentially inhibits anchorage-independent growth of cancer cells over anchorage-dependent growth (∼10 fold). It was also reported by others to have anti-tumor activity *in vivo* and selective toxicity against cancer cells under glucose starvation *in vitro*, but with unknown mechanism. Here, we provide evidence that pyrvinium suppresses the transcriptional activation of GRP78 and GRP94 induced by glucose deprivation or 2-deoxyglucose (2DG, a glycolysis inhibitor), but not by tunicamycin or A23187. Other UPR pathways induced by glucose starvation, *e.g.* XBP-1, ATF4, were also found suppressed by pyrvinium. Constitutive expression of GRP78 *via* transgene partially protected cells from pyrvinium induced cell death under glucose starvation, suggesting that suppression of the UPR is involved in pyrvinium mediated cytotoxicity under glucose starvation. Xenograft experiments showed rather marginal overall anti-tumor activity for pyrvinium as a monotherapy. However, the combination of pyrvinium and Doxorubicin demonstrated significantly enhanced efficacy *in vivo*, supporting a mechanistic treatment concept based on tumor hypoglycemia and UPR.

## Introduction

Intratumoral heterogeneity is a common characteristic of solid tumors [1]. As tumors progress and increase in size, cancer cells located in certain parts of the tumor interior, are confronted with hypoglycemia and hypoxia resulting from poor vascularization. The hypoglycemic state of cancer cells within solid tumors are usually further enhanced by the high energy demand met mainly through enhanced glycolysis, or the Warburg effect. Cancer cells under these stressful microenvironments become dormant and resistant to radiation and chemotherapy, while actively dividing cancer cells located at areas with sufficient blood supply, such as outer layers of tumors, are killed by these treatments [2], [3], [4]. Therefore, combination therapies that simultaneously attack both dormant hypoglycemic/hypoxic tumor cells and the proliferating tumor cells of solid tumors are likely to be more effective in tumor eradication than existing approaches.

Glucose-regulated protein 78 (GRP78) is a molecular chaperone resident in the endoplasmic reticulum (ER) and plays a major role in protecting cells from the apoptosis induced by ER stresses, including glucose deprivation and hypoxia [5], [6]. GRP78 and GRP94 (HSP90α), normally expressed at relatively low levels, are involved in facilitating folding, maturation and transportation of nascent proteins [5]. Under ER stress, the accumulation of unfolded or misfolded proteins in the ER triggers the unfolded protein response (UPR), marked by drastic induction of a number of UPR genes including GRP78 and GRP94 *via* transcriptional activation [7]. The UPR maintains cell survival by limiting cell proliferation and further accumulation of unfolded or misfolded proteins in ER. The UPR is initiated from dissociation of ATF6, PERK and IREα from GRP78 and subsequent transcriptional activation of UPR target genes. Studies in the recent decade have presented a complex network of signaling pathways activated by the UPR following ER stress, involving key proteins *e.g.* GRP78, GRP94, ATF-6, ATF-4, PERK, IREα, XBP-1 (Figure S1).

New evidence indicates that GRP78 plays a critical role in tumor development, progression and resistance to chemotherapy [5], [6]. Over-expression of GRP78 protein was found in a number of human tumors, *e.g.* aggressive breast cancer [8], [9], gastric, cancer [10], lung cancer [11], hepatocellular carcinoma [12] and prostate cancer [13]. GRP78 is also known to interact with caspase 7 to protect cells from apoptosis. Up-regulation of GRP78 appears to promote tumor growth and the increase of malignancy and drug resistance. The silencing of GRP78 by a small molecule was shown to be correlated to apoptosis under ER stress [14], [15]. Agents targeting the UPR pathway may provide a new strategy against cancer.

Pyrvinium pamoate was an old anthelminthic medicine. Esumi and colleagues recently reported that it was preferentially toxic to glucose-starved cancer cells and had anti-cancer activity in a hypovascular Panc-1 pancreatic cancer model, known to be resistant to hypoglycemia [16]. However, the mechanism of this anti-tumor activity has yet to be described. We recently independently identified pyrvinium pamoate from a small molecule compound library for preferential inhibiting anchorage-independent growth of cancer cells, and subsequently also confirmed the reported preferential toxicity to hypoglycemic cancer cells. We demonstrated that this effect can at least partially be attributed to its inhibition of the UPR. We explored a novel combination treatment scheme based on this mechanism of action of pyrvinium to enhance anti-tumor activity.

## Materials and Methods

### Compounds

Pyrvinium phosphate salt was prepared by following sequential steps: a) placing pyrvinium pamoate (ICN Pharmaceuticals, or Valeant) in an Erlenmeyer flask with a magnetic stirring bar, b) mixing with chloroform (Fisher), c) mixing with 95% ethanol (Pharmaco), d) warming to 50°C with stir for 10 minute, e) adding 2% phosphoric acid (85% in 95% ethanol) to produce a precipitate, f) adding ethyl acetate 2 minutes later, g) stirring for 20 minutes, h) collecting the solids by filtration followed by washing with 2/1/1 ethyl acetate/chloroform /ethanol, i) drying by air. The phosphate salt is freely soluble in water at 1 mg/ml, giving an orange red solution. 2-deoxyglucose (2DG), A23187 and tunicamycin were purchased from Sigma (Saint Louis, MO).

### Cells

A549, SW480, DLD1, A2058, MCF7, SK-BR-3, PA-1, DU145, PANC-1, IMR90, WI38, CCD-112CoN and HUVEC cells were purchased from American Tissue Culture Collection (ATCC) (Manassas, VA, USA) and cultured per recommendations. HOP62, HOP92, NCI-H460, NCI-H522, HCT116, HT29, HS 578T, T47D, MDA-MB-231, MDA-MB-435, A2780, OVCAR-1, OVCAR-4, OVCAR-5, OVCAR-8, SK-OV-3, Caov-3, UACC62, UACC257, and PC3 cells were obtained from NCI (Frederick, MD, USA) and were adapted to a single growth medium, RPMI 1640 (Invitrogen) supplemented with 10% FBS and 2 mM L-Glu. The establishment of C.1 cells and culture conditions for both CHO and C.1 cells were described previously[17]. All cells were maintained in a humidified incubator with 5% CO_2_ at 37°C.

### Plasmids, transfection and reporter gene assay

The pGL2-Grp78-169/LUC reporter gene was previously described [18]. Panc-1 cells of 40% confluence in 96-well plate format were co-transfected using Lipofectamine 2000 (Invitrogen) as previously described [19], with the same total amount of DNA containing pGL2-Grp78-169/LUC reporter gene plasmids and pRL Renilla luciferase expression plasmids, adjusted with pcDNA3 empty vector. The cells were harvested 24–36 hours post-transfection. The firefly luciferase activity was measured and normalized against *Renilla* luciferase activity using the Dual Luciferase kit (Promega) per protocol provided by manufacturer [20].

### Measurement of cell growth

Anchorage-dependent (liquid cell culture) and anchorage-independent (soft agar culture) growth in 96-well plates were previously described [19], [21]. For growth under glucose deprivation, the above anchorage-dependent growth in 96 well plates was modified as follows: cell suspensions were plated in each well of a 96-well flat-bottom microtiter plate on day 1 and treated with compounds at different concentrations in media with or without glucose on day 2. The cells were allowed to grow for three days before alamarBlue® staining (Trek Diagnostic Systems). Clonogenicity of CHO and C.1 cells was determined by the procedures described previously [17], [22].

For measurement of growth of the Panc-1 cells expressing exogenous GRP78, the cells in 96-well plates cells were co-transfected using Lipofectamine 2000 (Invitrogen) as with total same amount of DNA containing pcDNA-GRP78 and pGL2- LUC reporter gene plasmids, adjusted with pcDNA3 empty vector. The firefly luciferase activity was measured after transfection for 24 hrs as a surrogate of cell growth [20].

### Gene expression analysis

Real time RT-PCR used to quantitate mRNA levels in cells was described previously with PCR primer and probe sequences as follows: GRP78-probe: 5′-/56 FAM/CAAACTTGTCCCCGAGCGACGAGA /BHQ1/-3′, GRP78-forward primer: 5′- TGGCAGGAGAGAGTTACAGTCG, GRP78-reverse primer: 5′- GCCCTGCAGTCTCTCCCAC-3′); GRP94-probe: 5′-/56FAM/ TGTTTGACGAATA TGGATCTAAAAAGAGCGATTACAT/3BHQ-1/3′, GRP94-forward primer: 5′- CCCACATCTGCTCCACGTG; GRP94-reverse primer: 5′-CACGGCGCACATAG AGCTT -3′); ATF4-probe: 5′/56FAM/TTGGTCAGTCCCT CCAACAACAGC AAG /3BHQ-1/3′, ATF4-forward primer: 5′- TGGCTGGCTGT GGATGG; ATF4-reverse primer: 5′-TCCCGGAGAAGGCATCCT-3′); XBP1-probe: 5′-/56FAM/ CCCAGTTG TCACCCCTCCAGAACATCTC/3BHQ-1/3′, XBP1-forward primer: 5′- ACCTCTGCAGCAGGTGCAG; XBP1-reverse primer: 5′-AATACCGCCAGAATCCA TGG -3′). XBP1-spliced -probe: 5′-/56FAM/ CCCAGTTGTCACCCCTCCAGAACAT CTC /3BHQ-1/3′, XBP1-spliced -forward primer: 5′-ACCTCTGCAGCAGGTGCAG-3′; XBP1-spliced -reverse primer: 5′-AATACCGCCAGAATCCATGG -3′).

Western blot analysis procedure was also previously described[20], [21], [23]. Monoclonal antibody against KDEL and polyclonal antibody for HA tag were purchased from Stressgen Bioreagents (Ann Arbor, MI) and US Biological (Swampscott, Massachusetts) respectively. Polyclonal antibodies against ATF4, Erk2, phospho-Akt and Akt were obtained from Santa Cruz Biotechnology, Inc. (Santa Cruz, CA).

### Xenograft models

The establishment of human xenograft tumor was previously described [23]. Briefly, female athymic nude mice (*nu/nu*) (4–6 weeks old) were purchased from Simonsen Laboratory, Inc. (Gilroy, CA). The animal use and care protocol was approved by Perry Scientific's Animal Care and Use Committee (ACUC) and all procedures were conducted according to the guidelines of NIH/Department of Human and Health Services. Athymic mice were inoculated (*s.c.*) with 5×10^6^ cancer cells in PBS on the athymic mouse flanking site. Animals were randomly divided into treatment and control groups with similar average tumor sizes.

For HCT116 and AsPC-1 xenograft models, animals in the treatment group received pyrvinium pamoate daily at 10 mg/kg *via* oral administration (*p.o*.) and 0.5 mg/kg *via* intraperitoneal (*i.p.*) administration, 6 times weekly for five weeks respectively. For PC3 cancer model, animals in the first group were treated with pyrvinium pamoate daily at 10 mg/kg *via* oral dosing, 6 times weekly for five weeks. The second group was administrated with doxorubicin *i.p.* at 4 mg/kg, once per week for two weeks. The third group was treated with both pyrvinium pamoate and doxorubicin as described above. The control groups were dosed with 5% dextrose (vehicle) with the same schedule as the treatment group. Tumor volumes were manually measured and calculated as previously reported [23]. The significance of variability between the results of each group and its corresponding control was determined by ANOVA and independent Student's t-test. The level of statistical significance was set at 0.05.

## Results

### Pyrvinium broadly inhibits anchorage-independent growth of cancer cells

**Figure 1:**
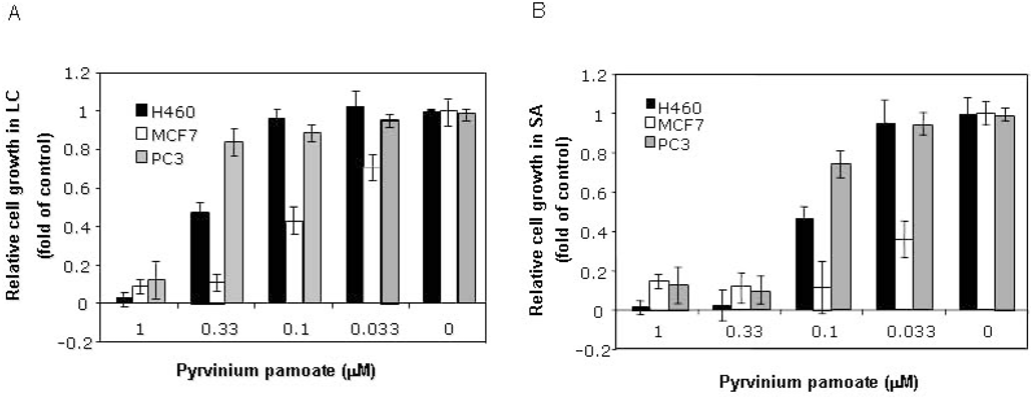
Sensitivity of different cancer cells to pyrvinium pamoate under anchorage -dependent and -independent conditions. Cells were seeded into each well of a 96-well plate in liquid culture (A) and soft agar culture (B) at 1e3/well for one day. Cells were treated with pyrvinium pamoate at indicated concentrations and cell growth in culture was measured after incubation at 37°C for 3 days (A) and 7 days by alamar Blue staining with excitation at 530±25 nm and emission at 590±35 nm (B). Error bars: standard deviations (SD).

Anchorage-independent growth of cancer cells is a hallmark of cell transformation. In an effort to identify compounds with selective anti-cancer activity, we screened a small compound library composed of drugs, drug-like and natural compounds, using 96-well format soft-agar (SA) assay, an anchorage-independent growth assay we recently developed [21]. Three NCI prototype cancer cell lines, PC3 prostate cancer, MCF-7 breast cancer and NCI-H460 NSCL, were used in our initial screen. Pyrvinium pamoate was identified among the hits with preferential inhibitory activity for anchorage-independent growth (Figure 1B) over anchorage-dependent growth (Figure 1A) measured by liquid culture (LIQ) growth. We then further examined pyrvinium pamoate against a large panel of cancer cells and confirmed this property in a majority of cancer cell lines tested (Table S1, Figure 1). The higher potency of pyrvinium pamoate against cancer cells in anchorage-independent growth suggests a possible desirable therapeutic index as cancer treatment.

During the course of our study, Esumi and colleagues reported anti-tumor activity of pyrvinium pamoate in human pancreatic cancer Panc-1 xenograft models [16]. More interestingly, they also described that pyrvinium pamoate sensitized cancer cells to glucose deprivation (IC_50_∼0.1 µM) or 3-dimensional growth (spheroid), both subsequently confirmed by us. Our soft agar growth assay may resemble spheroid growth since both are 3-dimensional without attaching to culture dish, although the interpretation for the observed sensitivity for spheroid growth by these authors was due to glucose deprivation inside the spheroid itself.

Pyrvinium pamoate is insoluble in water (<1 µM), thus not a practical salt form for *in vivo* pharmacological evaluation (see below). We created several new pyrvinium salts including phosphate and sulfate salts, with significantly increased water solubility (data not shown). These new salts were retested in the same *in vitro* assays and were confirmed to have the same phenotypes as seen for pyrvinium pamoate, verifying that the pyrvinium moiety, instead of pamoate, is responsible for the observed phenotypes since pamoate alone did not show any observable effect (Figure S2). Pyrvinium phosphate was used in all subsequent *in vitro* experiments, unless specified otherwise.

### Pyrvinium specifically suppresses the up-regulation of GRP78 and GRP94 induced by glycolytic inhibition

**Figure 2:**
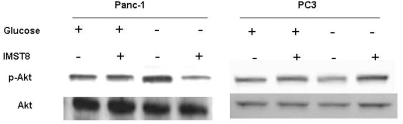
Effect of pyrvinium on Akt phosphorylation. Cells in culture media with or without glucose were treated with pyrvinium phosphate (pyrvinium-p) at 0.1 uM for 2 hrs. The phosphorylation levels of Akt were measured by western blot using anti-phospho-Akt and anti-Akt antibodies.

Esumi and colleagues reported that pyrvinium pamoate dephosphorylated Akt in Panc-1 cells only when glucose was deprived, which was correlated to the observed preferential cytotoxicity. This hinted a possible role of Akt in the pyrvinium pamoate action [16]. To verify whether this association generally exists in cancer cells, we compared Akt phosphorylation during glucose starvation and pyrvinium treatment in several additional human cancer cells. Although the preferential cytotoxcity remained in all the tested cells, the phosphorylation profiles of Akt varied greatly upon glucose deprivation and pyrvinium treatment as shown in Table 1 and Figure 2. This suggests that Akt is not a common mechanism for the observed biological effect of pyrvinium in many these cancer cells, although we cannot rule out the role of Akt in some of cells, *e.g.* panc-1. There must be other pathways involved in the pyrvinium actions.

**Table 1:** Akt dephosphorylation by pyvinium phosphate in various tumor cell lines under glucose starvation.

Cancer cell lines	Reduction of Phospho-Akt level
AsPC-1	Yes
PANC-1	Yes
A549	Yes
HCT116	No
PC3	No
JHMG	No
Hop62	No
H460	No

Note: These results are summary of Western blot analysis of cells cultured in growth media+/−glucose and treated with pyrvinium (0.1 µM, 2 hrs.), using anti-phospho-Akt and anti-Akt antibodies.

**Figure 3:**
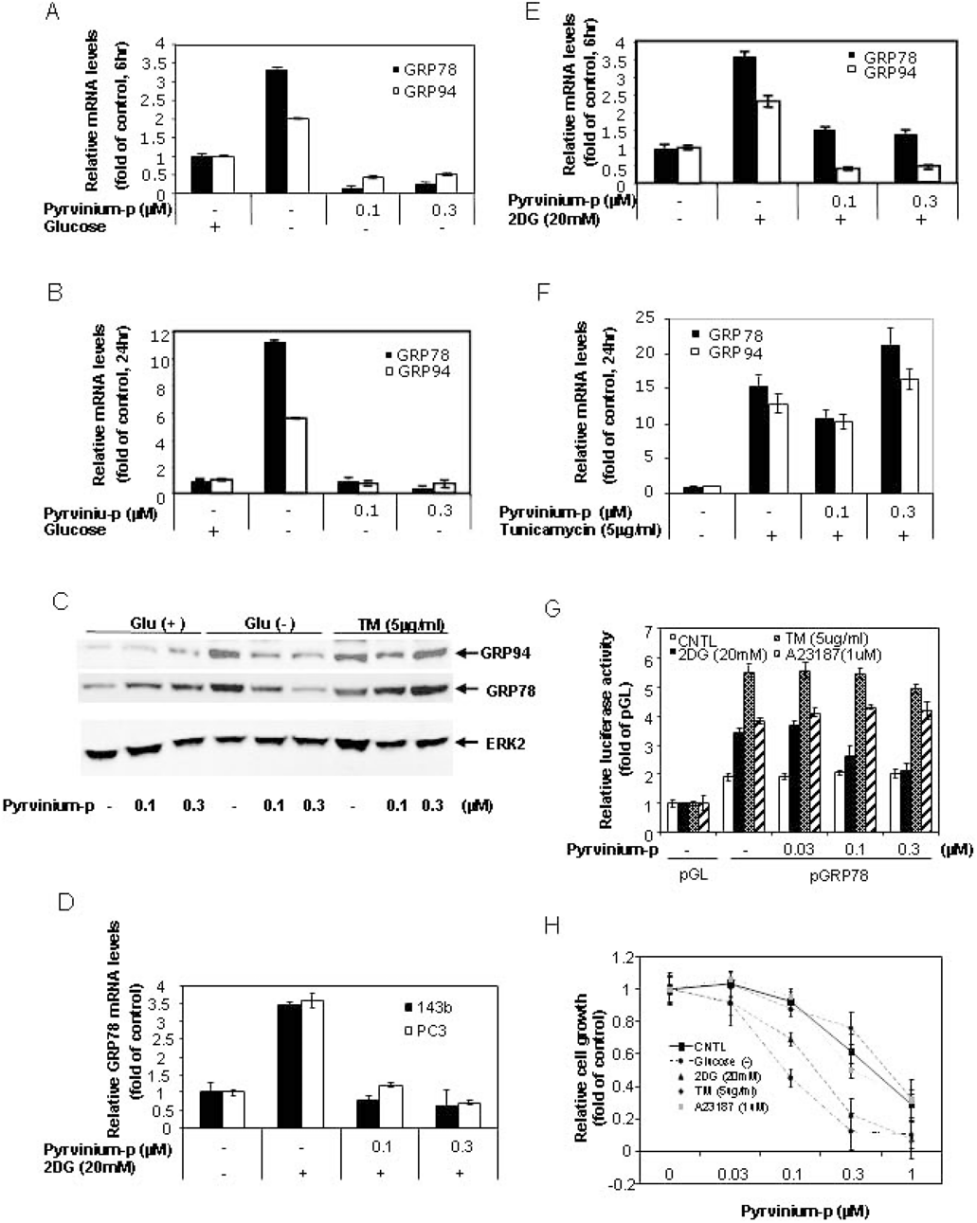
Pyrvinium suppresses GRP78 and GRP94 expression. Panel 3A, 3B, 3D 3E and 3F: Quantitative real-time RT-PCR analysis. Panc-1 cells (A, B, E and F) and PC3 and 143b cells (D), cultured in growth media with or without glucose (A and B), 2DG (D and E) and tunicamycin (F), were treated with pyrvinium phosphate (pyrvinium-p) for 6 hrs (A D and E) and 24 hrs (B and F). Total RNA was harvested and subjected to Taqman real-time PCR analysis for GRP78 and GRP94. Panel 3C: Western blot analysis. Panc-1 cells in growth media with or without glucose were treated with pyrvinium-p and tunicamycin for 24 hrs. The cell lysates were harvested for western blot using anti-KDEL antibody for detection of GRP78 and GRP94, Erk2 (internal control). Panel 3G: reporter gene assay for detection of GRP78 promoter. Panc-1 cells were co-transfected with GRP78-169/LUC and Renilla luciferase genes. The cells were treated with pyrvinium-p in combination with 2DG, tunicamycin or A23187 for 24 hrs. The firefly luciferase activity was measured and normalized against Renilla luiciferase activity using the dual luciferase kit. All of the transfections were performed in duplicates or triplicates. Panel 3H: cell growth assay. Panc-1 cells were treated with pyrvinium-p in combination with glucose starvation, 2DG, tunicamycin or A23187 for 72 hrs and cell proliferation was measured by alamarBlue staining (3 days in 96 well plates). Error bars: SD.

**Figure 4:**
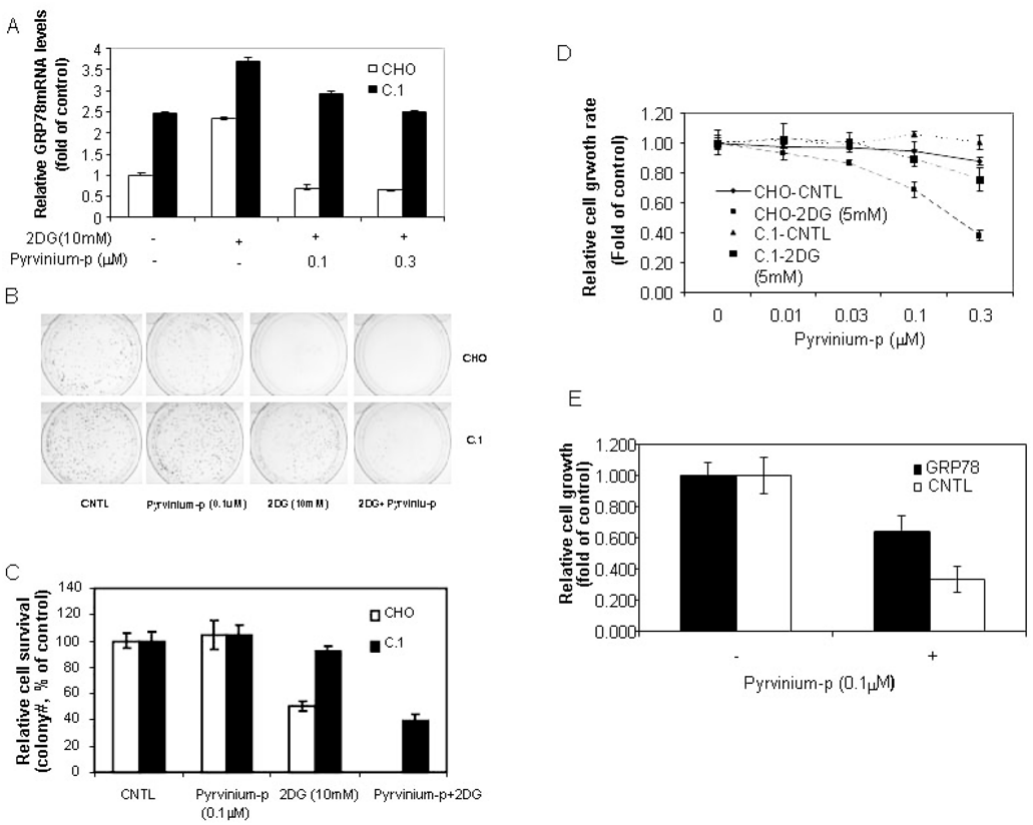
Over-expression of GRP78 protects cells from pyrvinium-induced cell death. A: Quantitative real-time RT-PCR analysis. CHO and C.1 cells cultured in growth media with or without 2DG were treated with pyrvinium phosphate (pyrvinium-p) for 6 hrs. Total RNA was harvested and subjected to Taqman real-time PCR analysis for GRP78. B and C: Clonogenicity assay. CHO and C.1 cells were seeded on day 1. The cultures were treated on day 3 with 2DG (10 mM), pyrvinium-p (0.1 µM) or combination of 2DG and pyrvinium-p for another 18 hrs and continued to culture for 2 weeks. The colonies were quantitated. D: alamarBlue readout of cell proliferation (3 days in 96 well plates) as described in figure 1. The SD of triplicate samples is shown.

GRP78 and GRP94 are two ER resident stress response chaperones that are transcriptionally induced during glucose deprivation as part of the UPR, which protects cells from apoptosis under a variety of stress conditions [5], [24]. We were therefore interested in whether pyrvinium affects GRP78 and GRP94 induction caused by glucose deprivation. Figures 3A and 3B shows the induction of GRP78 and GRP94 mRNA levels in Panc-1 cells, as measured by Taqman real-time RT-PCR for 6 hours (3 and 2 fold respectively) and 24 hours (11 and 5 fold respectively) of glucose starvation. Interestingly, these inductions were completely suppressed by pyrvinium treatments at 0.1 and 0.3 µM (Figure 3A and 3B), the concentrations causing cytotoxicity. Since there was no apparent cell death measured by alamarBlue assay for the 6 hour treatment, the observed suppression of the GRP78 and GRP94 induction did not result from cell death. Consistently, similar an inhibitory effect was also reflected at protein levels as shown by immunoblot analysis (Figure 3C). Importantly, this suppression was also seen in all the tested cells including PC3 human prostate cancer, 143b osterosarcoma cell lines (Figure 3D) and CHO cells (Figure 4), suggesting an ubiquitous effect of pyrvinium on the UPR

2-deoxyglucose (2DG) is a glucose analog, a glycolysis inhibitor and also a transcriptional activator of GRP78 or the UPR response [15], as seen in glucose starvation. We next investigated whether pyrvinium also suppressed GPR78 transcriptional activation by 2DG in Panc-1 cells. We found that the induction of GRP78 and GRP94 mRNA by 2DG was completely suppressed by pyvinium, similar to that seen in glucose deprivation (Figure 3E). There are other compounds also activating the UPR, which are all associated with the up-regulation of GRP78, including tunicamycin, an antibiotic inhibiting the first step of glycoprotein synthesis. Interestingly, pyrvinium did not suppress the GPR78/94 induction by tunicamycin (Figure 3F), demonstrating that the pyrvinium effect is limited to specific stress conditions of hypoglycemia and glycolysis inhibition.

We were also interested in whether down-regulation of the steady state levels of GRP78 mRNA/proteins resulted from transcriptional suppression, a characteristic UPR regulation. To this end, a reporter system for GRP78 transcription, pGL2-Grp78-169/LUC, was used. This system contains a GRP78 promoter driven luciferase reporter gene. The promoter harbors the *cis*-acting ER stress response element (ERSE) that is required for ER stress-mediated transcriptional activation [18], [25]. Since UPR induction can affect general protein synthesis including reporter protein synthesis, pGL2-Grp78-169/LUC was co-transfected along with a *renilla* luciferase gene driven by constitutive promoter as an internal control, which ensures that the observed effect is related to UPR-specific transcriptional regulation, rather than the changes in general protein synthesis or the variation in transfection efficiency. As shown in Figure 3G, Panc-1 cells transfected with pGL2-Grp78-169-LUC displayed induced luciferase activity by the treatments of 2DG, tunicamycin or calcium ionophore A23187 (another agent known to induce GRP78 [26]) (2∼3 fold over untreated) as reflected by the ratio of firefly to renilla luciferase activities. These promoter reporter activations are similar to the induced endogenous GRP78 mRNA levels, which confirms the transcriptional induction by these ER stresses. Pyrvinium selectively suppressed 2DG-induced, but not tunicamycin or A23187 induced GRP78 promoter reporter readouts in a dose-dependent fashion, confirming the transcriptional suppression of GRP78 by pyrvinium, which is specific to 2DG or glucose deprivation. These results suggest that pyrvinium likely targets the UPR pathway upstream of GRP78.

### Pyrvinium mediated GRP78 suppression contributes to the enhanced cytotoxicity under hypoglycemic conditions

Since GPR78/94 induction constitutes one of the key pro-survival mechanisms of cancer cells in response to glucose deprivation, pyrvinium-mediated suppression of GRP78 and GRP94 expression may counter this survival mechanism and resulted in rapid cell death. This assumption was indeed supported by the observed correlation between the suppressed expression of GRP78 and GRP94 by pyrvinium and cytotoxicity. As shown in Figure 3H, significantly increased cell death was observed when Panc-1 cells were treated with pyrvinium in combination with glucose starvation or 2DG, but not with tunicamycin and A23187. In order to verify that the suppression of GRP78 by pyrivinium indeed contributed to the enhanced cytotoxicity by pyrvinium in the presence of 2DG, we tested whether constitutively expression of GRP78 can reverse the pyrvinium induced cell death in the presence of 2DG. We previously described a GRP78 transgene expressing cell line C.1, a CHO line constitutively expressing exogenous GRP78 [17]. We tested C.1 by clonogenic assay in the presence of 2DG in parallel to the parental CHO cells. The result showed that C.1 cells, with exogenously expressed GRP78 that could not be down-regulated by pyrvinium (Figure 4A), were significantly more resistant to the treatments by 2DG, or by the combination of pyrvinium and 2DG, as compared to the parental CHO cells (Figure 4B and 4C). Similar results were also observed a cell proliferation assay in 96 well format (Figure 4D).

Although CHO cells, the parental cell line of C.1, also show the properties of preferential cytotoxicity and GRP78 down regulation by pyrivinium under glycolytic suppression as seen in Panc-1 and other tested cell lines (Figure 3D and Table S2), one may still argue that the rescued phenotype observed in C.1 cells may not be necessarily relevant to that in Panc-1 cells. To confirm this mechanism in panc-1 cells, we conducted a similar transient exogenous GRP78 over-expression experiment based on a luciferase-based survival assay that we previously developed [20]. This assay aimed to readily measure the effect of a gene on cell proliferation/survival using a transient transfection with enhanced signal/noise ratio. In this experiment, we co-transfected Panc-1 cells with the GRP78 expression construct and a SV40 promoter-driven luciferase expression cassette followed by measuring the luciferase activity as a surrogate marker for cell proliferation/survival of the cotransfected cells. Figure 4E demonstrates that ecotopic expression of GRP78 indeed resulted in significant resistance of cells to pyrvinium under glucose deprivation reflected by the higher luciferase activity than cells without ectopic expression of GRP78. Together, all these observations suggest that GPR78 down-regulation by pyrvinium indeed causally, at least in partial, contributes to the pyrvinium action leading to the enhanced cytotoxcity under glucose deprivation.

### Pyrvinium down-regulates spliced XBP-1

**Figure 5:**
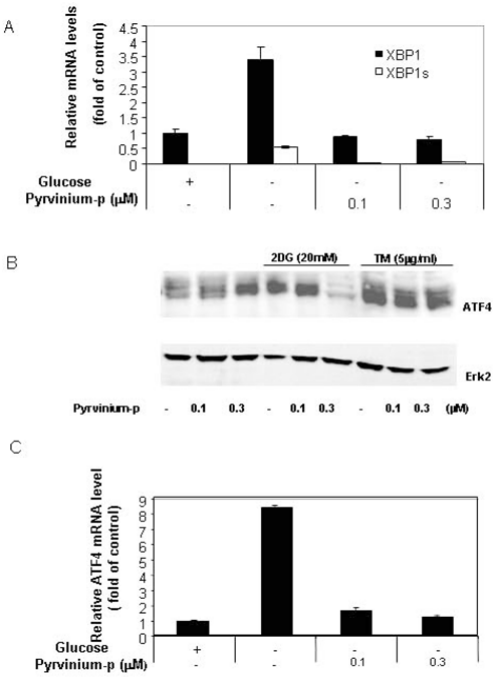
Pyrvinium suppresses XBP-1s, XBP-1 and ATF4 expression. 5A: Panc-1 cells cultured (±glucose) were treated with pyrvinium-p for 6 hrs. Taqman real-time PCR was conducted using primers and probes against XBP-1 (black bar) and XBP-1s (open bar). 5B: Western-blot analysis of the Panc-1 cells treated with 2DG, tunicamycin and pyrvinium for 24 hrs using anti-ATF4 and Erk2 antibodies (a representative of 3 independent experiments). 5C: Quantitative real-time RT-PCR analysis. Panc-1 cells cultured with or without glucose and pyrvinium treatment (24 hours) and the total RNA was harvested for detection of ATF4 mRNA levels. Error bars: SD.

Several factors are known to be responsible for ER stress-mediated transcriptional activation of GRP78, including the alternatively spliced XBP-1 (sXBP-1) and activated ATF6 [27]. We examined the effect of pyrvinium on XBP-1 splicing following glucose starvation to further understand its mechanism of action. Figure 5A shows that pyrvinium significantly down-regulates the induction of sXBP-1 mRNA by glucose deprivation, suggesting that XBP-1 is the more proximal target of pyrvinium than GRP78. Because the transcription of XBP-1 is also under ERSE control similar to GRP78, we examined the effect of pyrvinium on the expression of the total XBP-1 mRNA. As expected, pyrvinum also significantly suppressed the XBP-1 up-regulation (mRNA) by glucose starvation (Figure 5A). These results further support the notion that pyrvinium inhibits ERSE-dependent transcription mediated by glucose deprivation.

### Pyrvinium suppresses ATF-4 transcriptional activation by glucose starvation

ATF-4 is a cAMP response element-binding transcription factor (CREB) and promotes cell survival by activating certain gene transcription including those involved in the stress response, including the GRP78 promoter [28]. Previous data indicated that ATF-4 was activated by an alternative translational initiation in response to a general inhibition of protein synthesis as part of the UPR [29], [30], however, so far there has been no report on ATF-4 mRNA induction as part of UPR. However, when we investigated whether ATF-4 transcription is affected by pyrvinium, we found that 2DG or glucose starvation clearly up-regulated ATF4 at both protein (Figure 5B) and mRNA (Figure 5C) levels. The data provides evidence for the first time that ATF4 mRNA is also up-regulated by glucose starvation and tunicamycin likely as part of UPR, although it is still unclear whether this was due to increased transcription or mRNA stability. Further studies on ATF4 transcriptional regulation will give new insights into the UPR pathways.

With this new information of ATF-4 up-regulation by glucose deprivation, we next asked whether this up-regulation can also be suppressed by pyrvinium. We demonstrated that the mRNA and protein induction was completely suppressed by pyrvinium treatment. In contrast, no suppression was observed for tunicamycin-mediated ATF4 induction. These results again provided evidence that pyrvinium also affects the UPR by acting on the PERK pathway, another major UPR pathway. It is noteworthy that pyrvinium actually up-regulates ATF4 protein in the presence of glucose (Figure 5B), contrasting to that seen in the absence of glucose. Because ATF4 expression could be induced by other stresses unrelated to the UPR [31], pyrvinium may also trigger other stress pathways when glucose is available.

### Anti-tumor activity of pyrvinium alone is limited

**Figure 6:**
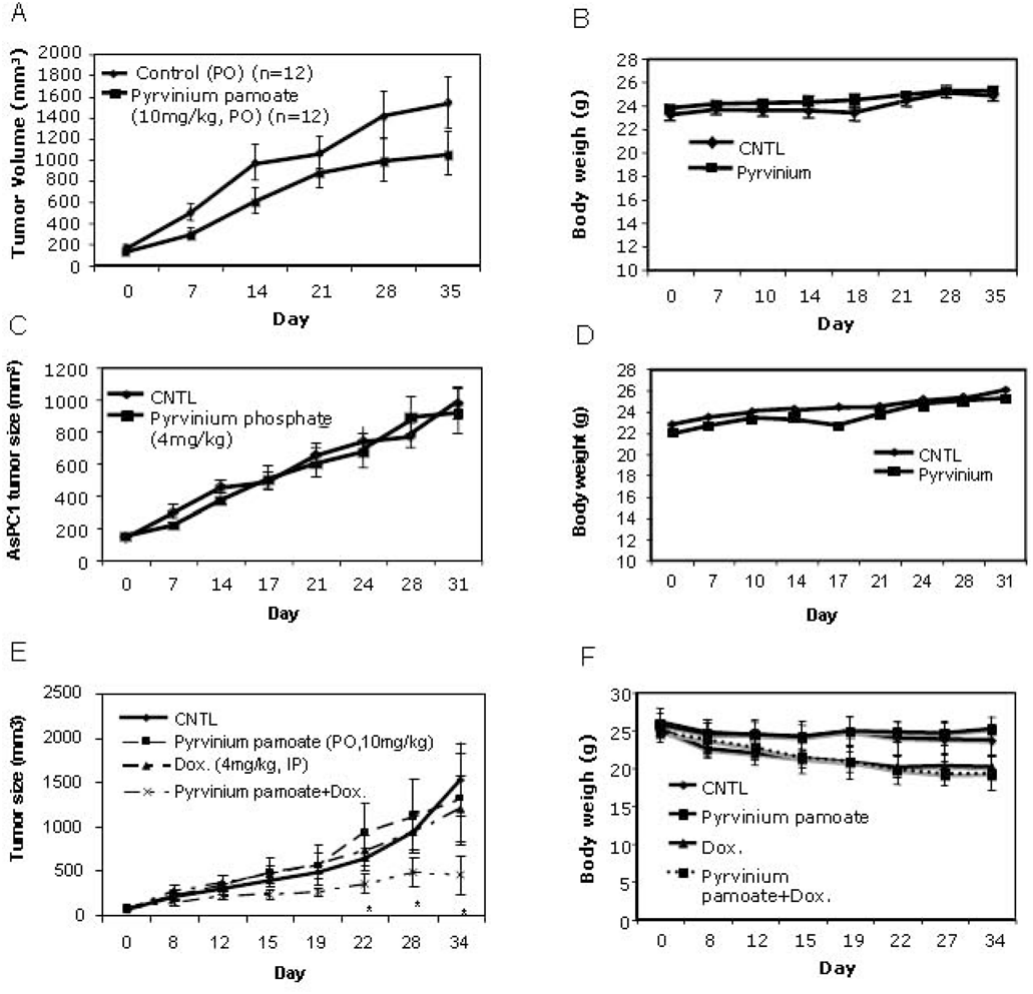
Anti-tumor effects of pyrvinium monotherapy or combination therapy with doxorubicin. 6A. 6B, 6C and 6D: HCT116 (A and B) and AsPC-1 (C and D) models. Pyrvinium pamoate was administrated either orally (*p.o.* 10 mg/kg) or *i.p.* (0.5 mg/kg) 6 times/week. 6E and 6F: pyrvinium pamoate combination therapy in PC3 model. Pyrvinium (*p.o.* 10 mg/kg 6 times/week), and/or Doxorubicin (*i.p.* 4 mg/kg, weekly for 2 weeks) were administrated. 10 or 12 mice in each group (n = 10 or 12) were used for all the experiments. Error bars: standard error (SE); *: p<0.05.

On the basis of the higher potency against cancer cells deprived of glucose, Esumi and colleagues reasoned that pyrvinium pamoate could potentially be effective against solid tumors, since parts of solid tumors are usually deprived of glucose, oxygen and other nutrients. They tested this hypothesis by assessing anti-tumor activity in a Panc-1 pancreatic cancer xenograft model, since pancreatic cancers are poorly vascularized (with less access to glucose) and insensitive to hypoglycemia, as compared to other solid tumors. They confirmed their hypothesis by demonstrating the Panc-1 xengraft tumor response to oral administration of pyrvinium pamoate [16]. In order to assess breadth of pyrvinium anti-solid tumor activity, we tested pyrvinium pamoate and other soluble pyrvinium salts in several additional xenograft models, either *via* oral administration or *i.p.* injection. We tested systemic delivery of soluble pyrvinium salt to enhance anti-tumor activity by overcoming the limitation of oral absorption. Overall, our data showed rather marginal tumor responses to pyrvinium treatment (see Table S3, Figure 6A and 6B), with some responses seen in HCT116 colon cancer and A2780 ovarian cancer (early staged tumors) models, but little in PC3 prostate cancer, AsPC1 pancreatic cancer, and A549 NSCL tumor models. Furthermore, when *i.p.* dosed with the soluble salts, the dose-limiting toxicity was clearly evident, contrasting to the safe oral administration of pyrvinium regardless of salt form and confirming the poor oral absorption of pyrvinium.

### Combination therapy of pyrvinium with doxorubicin is significantly more effective than monotherapy

The weak anti-tumor activity of pyrvinium could be explained by the following possibilities: first, attacking the hypoglycemic tumor areas is simply insufficient to mount an effective overall anti-cancer treatment, similar to those seen in many anti-angiogenic monotherapies; second, glucose levels in tumors are still too high to render pyrvinium effective. These two putative limitations can potentially be overcome by combination therapy with existing chemotherapy agents that efficiently kill the dividing cancer cells in normal glycermic areas (*e.g.* exterior) and/or further deprive glucose levels in solid tumor *via* shutting down blood flow, thus broadening therapeutic window. We therefore evaluated the combination therapy of pyrvinium with a conventional chemotherapy agent doxorubicin for being potent against dividing cancer cells and their anti-angiogenic effects.

A PC3 xenograft cancer model was employed for the doxorubicin combination treatment since PC3 tumors treated by doxorubicin had been well documented with detailed dosing, toxicity and efficacy information [32], which served as guidance for the first combination experimental design. In addition, both doxorubicin and pyrvinium are rather ineffective against PC3 xenograft tumor growth, thus an idea model for testing combination. We established 50 PC3 tumor xenografts in athymic mice and allowed the tumors to reach average volumes of 150 mm^3^. The tumor-bearing animals were then divided into four treatment groups: group 1 (PBS daily oral (*p.o.*) dosing), group 2 (4 mg/kg doxorubicin once a week *i.p.* dosing for 2 week), group 3 (10 mg/kg pyrvinium daily oral (*p.o.*) dosing), group 4 (4 mg/kg doxorubicin once a week *i.p.* dosing for 2 weeks with 10 mg/kg pyrvinium daily oral (*p.o.*) dosing). The tumor responses to these treatments are shown in Figure 6E. While little response was seen for either single agent treatments of doxorubicin and pyrvinium phosphate, significant response to their combination was observed, as compared to control (p<0.05). This demonstrated the likely synergistic inhibition of PC3 tumor growth by the pyrvinium and doxorubicin combination *in vivo*. General toxicity was evaluated on the basis of changes in animal body weight (Figure 6B, 6D and 6F). Among all the treated groups, only animals that received doxorubicin only or the combination therapy showed the similar body weight loss (∼20%, Figure 6F), suggesting that the observed toxicity is due to doxorubicin but not pyrvinium.

## Discussion

Esumi and colleagues proposed a possible role for Akt in the action of pyrvinium pamoate, since pyrvinium inhibits Akt phosphorylation in glucose deprived Panc-1 cells [16]. However, several observations suggest that this pathway may not be essential or universal. First, LY294002, a PI3K inhibitor, did not abolish Panc-1 cell tolerance to glucose deprivation even though it inactivated Akt as shown by the same group [33] and by our own data (not shown). Second, pyrvinium pamoate dephosphorylated Akt that was induced by glucose starvation, but failed to inactivate Akt activity induced by insulin [16]. Third, diverse cancer cell lines exhibited highly varied Akt expression profiles with pyrvinium treatment under glucose deprivation, which are not correlated to the consistent survival phenotypes (Figure 2 and Table 1). Therefore, while Akt might have been involved in specific cancer cell lines, other additional mechanisms are also likely involved in pyrvinium action.

Our data demonstrated that pyrvinium specifically suppressed the transcriptional induction of UPR activators by glucose deprivation, including GRP78, XBP1 and ATF4. These activities are consistent in all the tested cells and correlated with the selective toxicity of pyrvinium to glucose-deprived cells, contrasting to that of Akt, suggesting a possible novel mechanism for pyrvinium. The fact that over-expression of exogenous GRP78 increased the resistance to pyrvinium under glucose deprivation confirmed the causal link between UPR suppression and pyrvinium action.

The UPR is a protective mechanism by tumor cells adapting to stressful microenviroments, *e.g.* hypoxia, nutrient deprivations and the exposure to genotoxic agents. Shutting-down UPR induction sensitizes cells to these stresses. Versipelostatin, a small molecule, was recently reported to selectively suppress UPR induction by glucose deprivation ([15] also see Figure S3 and Table S4). It also has anti-tumor activity in xenograft tumor models, further supporting the notion that suppressing glucose starvation mediated UPR could be a potential novel anticancer strategy. Pyrvinium displays some similarity to that of versipelostain (Table S4). Both compounds seem to target upstream pathways of GRP78, but likely *via* different molecular targets. The difference is reflected by the facts that first, versipelostain mainly causes apoptosis to glucose deprived cancer cells [14], [15], while pyrvinium also triggers necrosis [16] with significantly higher potency (IC_50_: 0.03–0.1 µM *vs.* 1–3 µM) in cytotoxicity and inhibition of GRP78 (10∼100 fold); second, there absolutely is no structural similarity between the two (Figure S3); third, while UPR has been demonstrated to be causally linked to pyrvinium action, the similar causal role of UPR for versaipelostain action has yet been reported.

The existing chemotherapies are limited by two major factors: narrow therapeutic window and rapid development of drug resistance. The narrow therapeutic window is largely reflected by the low selectivity against cancerous tissues versus normal tissues. Pyrvinium has unique selectivity against hypoglycemic tumor tissues *vs.* normal tissues under normal glucose condition. This selectivity likely results from its impacts on the transformation pathways relevant to energy metabolism, and can thus be exploited to increase the therapeutic window. The development of drug resistance is complex and involves diverse mechanisms, one of which, in solid tumor therapy, is hypoxia/ hypoglycemia in the tumor interior resulting from hypovasculation [34]. An agent, such as pyrvinium, specifically targeting the drug resistant and dormant solid tumor interior will have particular therapeutic value.

Our attempted pyrvinium combination therapy with toxic agents/anti-angiogenic agent (Doxorubicin) was proven to be effective, supporting our assumed effective solid tumor treatment mechanism based on the simultaneous targeting of hypoglycemic areas and normal glycermic areas. One of the most serious problems confronting current chemotherapies is their undesirable general toxicity, *e.g.* the life-dose limitation of Doxorubicin [35]. The enhancement of efficacy (Doxorubicin), as shown in this study, suggests that the combination treatment may have safer and more practical advantages over the single chemotherapy in the current clinical settings.

## Supporting Information

Figure S1 ER stress-mediated UPR signal pathways. Red color represents targets or pathways affected by both pyrvinium and versipelostatin.
(4.51 MB TIF)

Figure S2 Inhibition of cell growth by different pyrvinium salts. Cells were seeded into each well of a 96-well plate in liquid culture with or without glucose at 1e3/well for one day. Cells were treated with pyrvinium at indicated concentrations and cell growth in culture was measured after incubation at 37°C for 3 days by alamarBlue staining. Error bars: standard deviations (SD).
(2.78 MB TIF)

Figure S3 Structures of pyrvinium and versipelostatin.
(2.31 MB TIF)

Table S1 Pyrvinium phosphate preferentially inhibits cancer cell anchorage-independent growth over anchorage-dependent growth
(0.06 MB DOC)

Table S2 Pyrvinium phosphate preferentially inhibits cancer cell growth deprived with glucose
(0.03 MB DOC)

Table S3 A Summary of tumor responses to pyrvinium in xenograft tumor models
(0.05 MB DOC)

Table S4 Comparison of pyrvinium and VST-1 effects on UPR
(0.03 MB DOC)
