# Supplementary figures and images for: Pyrvinium Targets the Unfolded Protein Response to Hypoglycemia and Its Anti-Tumor Activity Is Enhanced by Combination Therapy
Source: PLoS One. 2008 Dec 16;3(12):e3951. doi: 10.1371/journal.pone.0003951 (PMC2597738; doi:10.1371/journal.pone.0003951)

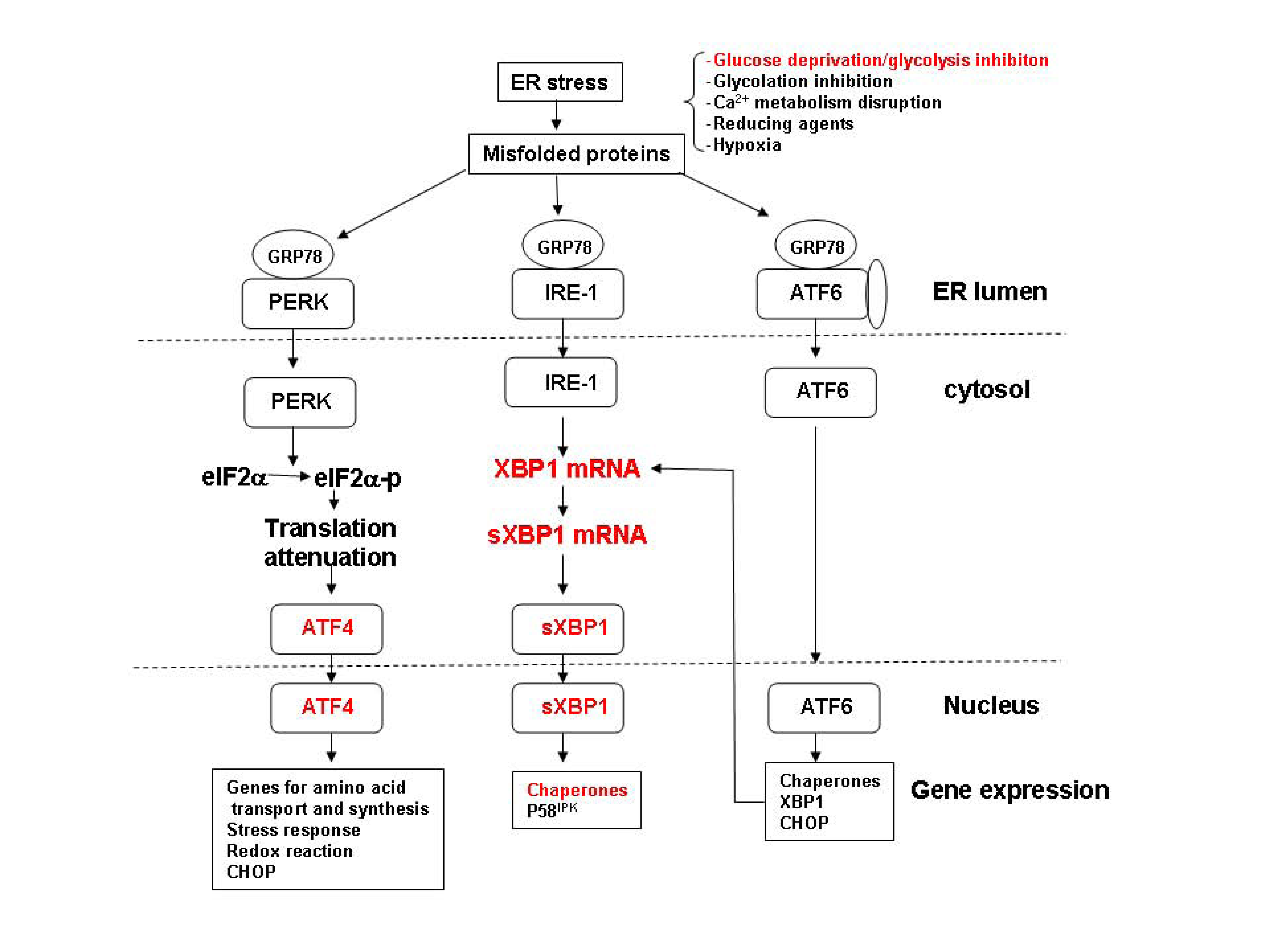

Supplement: Figure S1 — ER stress-mediated UPR signal pathways. Red color represents targets or pathways affected by both pyrvinium and versipelostatin. (4.51 MB TIF) [file pone.0003951.s001.tif]

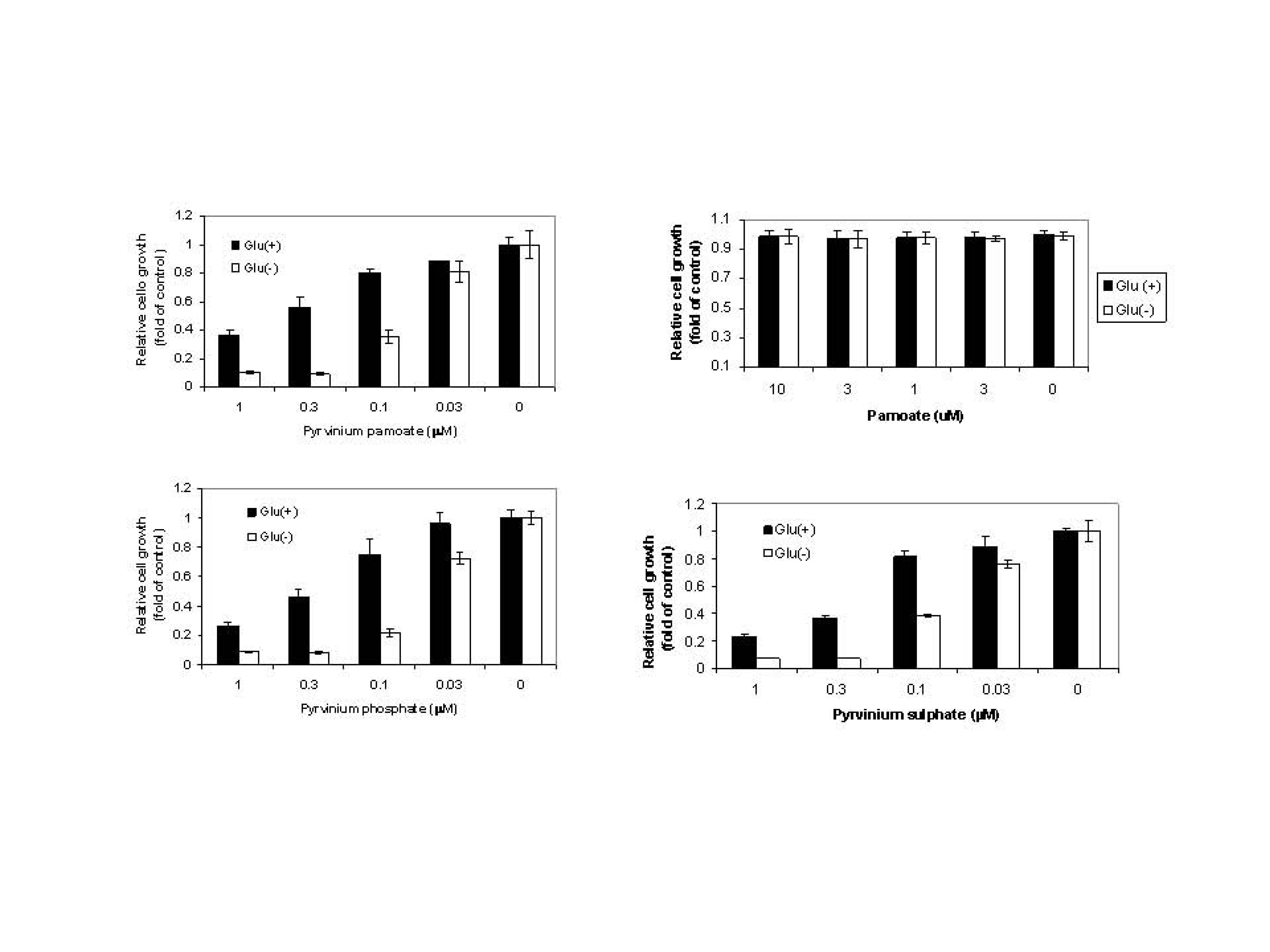

Supplement: Figure S2 — Inhibition of cell growth by different pyrvinium salts. Cells were seeded into each well of a 96-well plate in liquid culture with or without glucose at 1e3/well for one day. Cells were treated with pyrvinium at indicated concentrations and cell growth in culture was measured after incubation at 37°C for 3 days by alamarBlue staining. Error bars: standard deviations (SD). (2.78 MB TIF) [file pone.0003951.s002.tif]

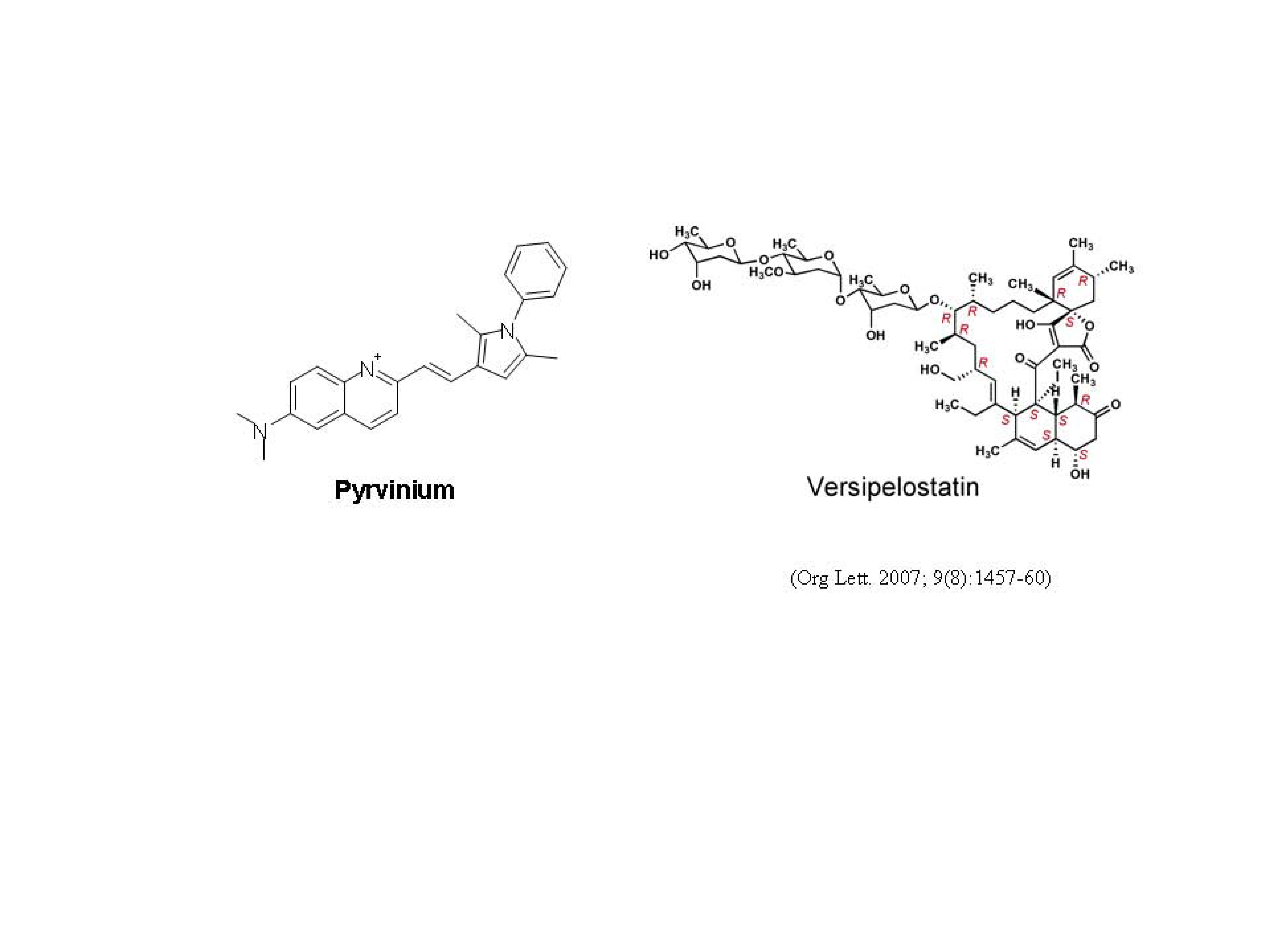

Supplement: Figure S3 — Structures of pyrvinium and versipelostatin. (2.31 MB TIF) [file pone.0003951.s003.tif]
