# Supplementary material for: Pyrvinium Targets the Unfolded Protein Response to Hypoglycemia and Its Anti-Tumor Activity Is Enhanced by Combination Therapy
Source: PLoS One. 2008 Dec 16;3(12):e3951. doi: 10.1371/journal.pone.0003951 (PMC2597738; doi:10.1371/journal.pone.0003951)
Supplement: Table S1 — Pyrvinium phosphate preferentially inhibits cancer cell anchorage-independent growth over anchorage-dependent growth (0.06 MB DOC) [file pone.0003951.s004.doc]

**Table S1. Pyrvinium phosphate preferentially inhibits cancer cell anchorage-independent growth over anchorage-dependent growth**

| Cell type | Cell line | IC_50_(Liq) | IC_50_(SA) |
| --- | --- | --- | --- |
| Breast C. | MCF7 | 0.03-0.1 | <0.03 |
|  | T47D | ~0.03 | <0.03 |
|  | SKBR3 | ~1 | ~1 |
|  | MDA-231 | 0.1-0.3 | <0.03 |
|  | MDA_MB-435 | 0.3-1 | 0.03-0.1 |
|  | HS578 | 0.1-0.3 | 0.03-0.1 |
| Colon C. | HCT116-luc | >1 | 0.1-0.3 |
|  | HCT116-p53si | >1 | 0.1-0.3 |
|  | HT29 | >1 | 0.03-0.1 |
|  | SW480 | 0.3-1 | 0.3-1 |
|  | DLD1 | 0.3-1 | ~1 |
| Prostate C. | PC3M/N | >1 | ~0.3 |
|  | PC3 | 0.1-0.3 | 0.1-0.3 |
|  | DU145 | >1 | 0.3-1 |
| Ovarian C. | A2780 | 0.03-0.1 | 0.03-0.1 |
|  | OVCAR-1 | 0.03-0.1 |  |
|  | PA-1 | 0.03-0.1 |  |
|  | OVCAR-8 | 0.1-0.3 | 0.03-0.1 |
|  | SK-OV-3 | 0.1-0.3 | 0.03-0.1 |
|  | OVCAR-4 | 0.1-0.3 |  |
|  | OVCAR-5 |  | 0.03-0.1 |
|  | Caov-3 | 0.03-0.1 |  |
| NSCL | A549 | 0.1-0.3 | 0.033-0.1 |
|  | NCI-H460 | 0.1-0.33 | 0.03-0.1 |
|  | HOP-62 |  |  |
|  | HOP-92 |  |  |
|  | NCI-H522 |  |  |
| Melanoma | UACC62 | 0.1-0.3 | 0.03-0.1 |
|  | UACC257 | ~0.1 | 0.1-0.3 |
|  | A2058 | 0.1-0.3 |  |
| Normal | HUVEC | 0.03-0.1 |  |
|  | W138 | 0.1-0.3 |  |
|  | IMR90 | 0.1-0.3 |  |
|  | CCD112/N | ~1 |  |

Note: All samples were performed in triplicate; and the average concentration

of 50% of growth inhibition (IC_50_) was presented.
