# Supplementary material for: Pyrvinium Targets the Unfolded Protein Response to Hypoglycemia and Its Anti-Tumor Activity Is Enhanced by Combination Therapy
Source: PLoS One. 2008 Dec 16;3(12):e3951. doi: 10.1371/journal.pone.0003951 (PMC2597738; doi:10.1371/journal.pone.0003951)
Supplement: Table S2 — Pyrvinium phosphate preferentially inhibits cancer cell growth deprived with glucose (0.03 MB DOC) [file pone.0003951.s005.doc]

**Supplement Table 2. Pyrvinium phosphate preferentially inhibits cancer cell growth**

**deprived with glucose**

| Cell type | Cell line | IC_50_(glu +) | IC_50_(glu-) |
| --- | --- | --- | --- |
| Breast C. | MDA-231 | 0.3-1.0 | 0.03-0.1 |
| Colon C. | HCT116 | 0.3-1.0 | 0.03-0.1 |
| Prostate C. | PC3M/N | 0.3-1.0 | 0.03-0.1 |
| NSCL | A549 | 0.1-0.3 | 0.03-0.1 |
| Pancreatic C. | PANC-1 | 0.1-0.3 | 0.03-0.1 |
|  | ASPC-1 | 0.3-1.0 | 0.03-0.1 |
| Glioma | U87 | 0.1-0.3 | 0.03-0.1 |
| Liver C | HepG2 | 0.3-1.0 | 0.1-0.3 |
| Osterosarcoma | 143b | 0.3-1.0 | 0.03-0.1 |
