# Supplementary material for: Pyrvinium Targets the Unfolded Protein Response to Hypoglycemia and Its Anti-Tumor Activity Is Enhanced by Combination Therapy
Source: PLoS One. 2008 Dec 16;3(12):e3951. doi: 10.1371/journal.pone.0003951 (PMC2597738; doi:10.1371/journal.pone.0003951)
Supplement: Table S3 — A Summary of tumor responses to pyrvinium in xenograft tumor models (0.05 MB DOC) [file pone.0003951.s006.doc]

**Table S3. A Summary of tumor responses to pyrvinium in xenograft tumor models**

| **Model** | **Compounds** | **Cancer type** | **Tumor response**  **(Inhibition, %)** | **Route** | **Comments** |
| --- | --- | --- | --- | --- | --- |
| PANC-1 | Pyrvinium pamoate | Pancreatic | 60-70 | Oral | Esumi et al |
| AsPC-1 | Pyrvinium phosphate | Pancreatic | <20 | *i.p.* | Figure 5 |
| HCT116 | Pyrvinium pamoate | Colon | 20-40 | Oral | Figure 5 |
| PC3 | Pyrvinium pamoate | Prostate. | <20 | Oral | Figure 5 |
| PC3 | Dox. | Prostate | 20-40 |  | Figure 5 |
| PC3 | Pyrvinium pamoate +Dox. | Prostate | 60-70 | Oral | Figure 5 |
| PC3 | Taxol | Prostate | 60-70 | *i.v.* | Data not shown |
| PC3 | Pyrvinium  pamoate +Taxol | Prostate | >80 |  | Data not shown |
| A549 | Pyrvinium phosphate | NSCL | <20 | *i.p.* | Data not shown |
| A2780 | Pyrvinium phosphate | Ovarian | 20-40 | *i.p.* | Earlier stage tumor and data not shown |

Note: NSCL, non-small cell lung cancer; % response =100X(C-T)/C.

**Table S4**. Comparison of pyrvinium and VST-1 effects on UPR

|  | Pyvinium | VST-1* |
| --- | --- | --- |
| GPR78 | Yes | Yes |
| GPR94 | Yes | Yes |
| XBP-1 | Yes | Yes |
| sXBP-1 | Yes | Yes |
| ATF-6 | NT | No |
| ATF-4 | Yes | Yes |
| Cell proliferation IC_50_ | 0.03~0.1μM | 1~3μM |
| GPR78 over expression | Rescued | NT |

Note: Yes: inhibitory effect; No: no effect; NT: not tested; *: Park et al., 2004.
