# Supplementary material for: Pyrvinium Targets the Unfolded Protein Response to Hypoglycemia and Its Anti-Tumor Activity Is Enhanced by Combination Therapy
Source: PLoS One. 2008 Dec 16;3(12):e3951. doi: 10.1371/journal.pone.0003951 (PMC2597738; doi:10.1371/journal.pone.0003951)
Supplement: Table S4 — Comparison of pyrvinium and VST-1 effects on UPR (0.03 MB DOC) [file pone.0003951.s007.doc]

**Table S4**. Comparison of pyrvinium and VST-1 effects on UPR

|  | Pyvinium | VST-1* |
| --- | --- | --- |
| GPR78 | Yes | Yes |
| GPR94 | Yes | Yes |
| XBP-1 | Yes | Yes |
| sXBP-1 | Yes | Yes |
| ATF-6 | NT | No |
| ATF-4 | Yes | Yes |
| Cell proliferation IC_50_ | 0.03~0.1μM | 1~3μM |
| GPR78 over expression | Rescued | NT |

Note: Yes: inhibitory effect; No: no effect; NT: not tested; *: Park et al., 2004.
